# *Pseudomonas aeruginosa* tRNA nucleotidyltransferase Cca controls resistance and tolerance to aminoglycoside antibiotics by regulating the MexXY multidrug efflux pump

**DOI:** 10.1128/aac.01653-25

**Published:** 2026-03-03

**Authors:** Chenyu Shen, Lepeng Wang, Yu Zhang, Linyan Zhang, Zhihui Cheng, Weihui Wu, Un-Hwan Ha, Shouguang Jin, Yongxin Jin

**Affiliations:** 1 State Key Laboratory of Medicinal Chemical Biology, Key Laboratory of Molecular Microbiology and Technology of the Ministry of Education, Department of Microbiology, College of Life Sciences, Nankai University 12538, Tianjin, China; 2 Department of Biotechnology and Bioinformatics, Korea University 34973, Sejong, Republic of Korea; University of Fribourg, Fribourg, Switzerland

**Keywords:** *Pseudomonas aeruginosa*, tRNA nucleotidyltransferase Cca, MexXY, aminoglycoside antibiotics, ArmZ

## Abstract

*Pseudomonas aeruginosa* displays high resistance to a wide range of antibiotics, greatly impeding clinical treatment. MexXY efflux pump plays an important role in bacterial resistance and tolerance to aminoglycoside antibiotics. tRNA nucleotidyltransferase (Cca, CCA-adding enzyme) is a universal polymerase responsible for the synthesis and repair of the 3′-terminal CCA sequence of tRNA. In this study, we found that mutation of the *cca* gene increases resistance and tolerance to aminoglycosides in *P. aeruginosa*. We demonstrate that the upregulation of *mexXY* genes contributes to the increased resistance and tolerance of the Δ*cca* mutant. Furthermore, our experimental results revealed that Cca controls the transcription of *armZ*, which encodes a positive regulator of *mexXY*, through its leader peptide PA5471.1. An increased amount of *PA5471.1* mRNA was found to be associated with ribosomes in the Δ*cca* mutant, indicating enhanced ribosome stalling. In addition, we found that the glutamine and phenylalanine at the third and fourth codons of PA5471.1, respectively, are involved in the Cca-mediated transcriptional upregulation of *armZ*. Therefore, our data reveal the molecular mechanism of Cca-mediated regulation of aminoglycoside susceptibility in *P. aeruginosa*.

## INTRODUCTION

*Pseudomonas aeruginosa* is a gram-negative opportunistic human pathogen that can cause various acute and chronic infections in cystic fibrosis patients, immunocompromised individuals, and burn victims (1). It has intrinsic resistance and easily acquires resistance to a wide range of antibiotics, posing a great challenge to clinical treatment (2). *P. aeruginosa* possesses multiple mechanisms to defend against antibiotics, including multidrug efflux systems that export antimicrobial drugs outside bacterial cells.

Four multidrug efflux systems associated with resistance to antibiotics have been well elucidated in *P. aeruginosa*, including MexAB-OprM, MexCD-OprJ, MexEF-OprN, and MexXY-OprM (3). MexXY is an inducible efflux system that contributes to the bacterial resistance to aminoglycosides (4–6). Expression of the *mexXY* is regulated by its repressor MexZ, encoded by the immediately upstream gene *mexZ* (7). MexZ binds to the intergenic region of *mexX-mexZ* to repress expression of *mexXY* and *mexZ* itself (7, 8). PA5471, also named ArmZ for anti-repressor MexZ, functions as an anti-repressor by binding to the MexZ and reducing its DNA-binding ability (7, 9). Expression of *armZ* is induced by antibiotics, mediated by its leader peptide-encoding gene *PA5471.1* upstream of the *armZ* (10). In the presence of ribosome-targeting antibiotics, such as aminoglycosides, the ribosome stalls on the mRNA of *PA5471.1*, altering the RNA secondary structure and leading to the transcription of *armZ* and, ultimately, *mexXY*, thereby conferring bacterial resistance to aminoglycosides (10).

tRNAs are adapter molecules that translate the genetic codes of mRNA into protein sequences. To be charged with their cognate amino acids, mature tRNAs carry the universally conserved CCA sequence at their 3′ termini (11). tRNA nucleotidyltransferase, also known as CCA-adding enzyme, is responsible for the synthesis and repair of the 3′-terminal CCA sequence (12). Most organisms rely on the posttranscriptional addition of the CCA triplet mediated by CCA-adding enzymes (13). However, some bacteria, like *Escherichia coli*, carry the CCA triplet in their tRNA genes (13). Therefore, acting as a tRNA repair enzyme, inactivation of the CCA-adding enzyme is not lethal for *E. coli* but only decreases the growth rate (14). While the function and structure of the CCA-adding enzyme in tRNA modification have been characterized (13, 15–17), other roles of the Cca in bacteria have not yet been elucidated.

Previously, we demonstrated that the ribosome-associated protein SuhB controls susceptibility to aminoglycoside antibiotics by modulating ribosome stalling and consequent expression of the MexXY multidrug efflux pump in *P. aeruginosa* (18). Polynucleotide phosphorylase (PNPase) controls bacterial tolerance to aminoglycosides by influencing the translation of *armZ* and thereby regulating MexXY (8). In this study, we found that mutation of the *cca* gene resulted in increased resistance and tolerance to aminoglycosides in *P. aeruginosa*. MexXY is upregulated in the Δ*cca* mutant, which contributes to the increased resistance and tolerance to aminoglycosides. We further demonstrated that *PA5471.1* is involved in the upregulation of *armZ* in the Δ*cca* mutant. An increased amount of *PA5471.1* mRNA was found to be associated with ribosomes purified from the Δ*cca* mutant, indicating enhanced ribosome stalling. Furthermore, we found that the glutamine and phenylalanine at the third and fourth codons of PA5471.1, respectively, are involved in the Cca-mediated transcriptional upregulation of *armZ* and consequent upregulated expression of the *mexXY* efflux pump, as well as resistance and tolerance to aminoglycoside antibiotics.

## RESULTS

### Cca influences bacterial resistance and tolerance to aminoglycosides

**Fig 1:**
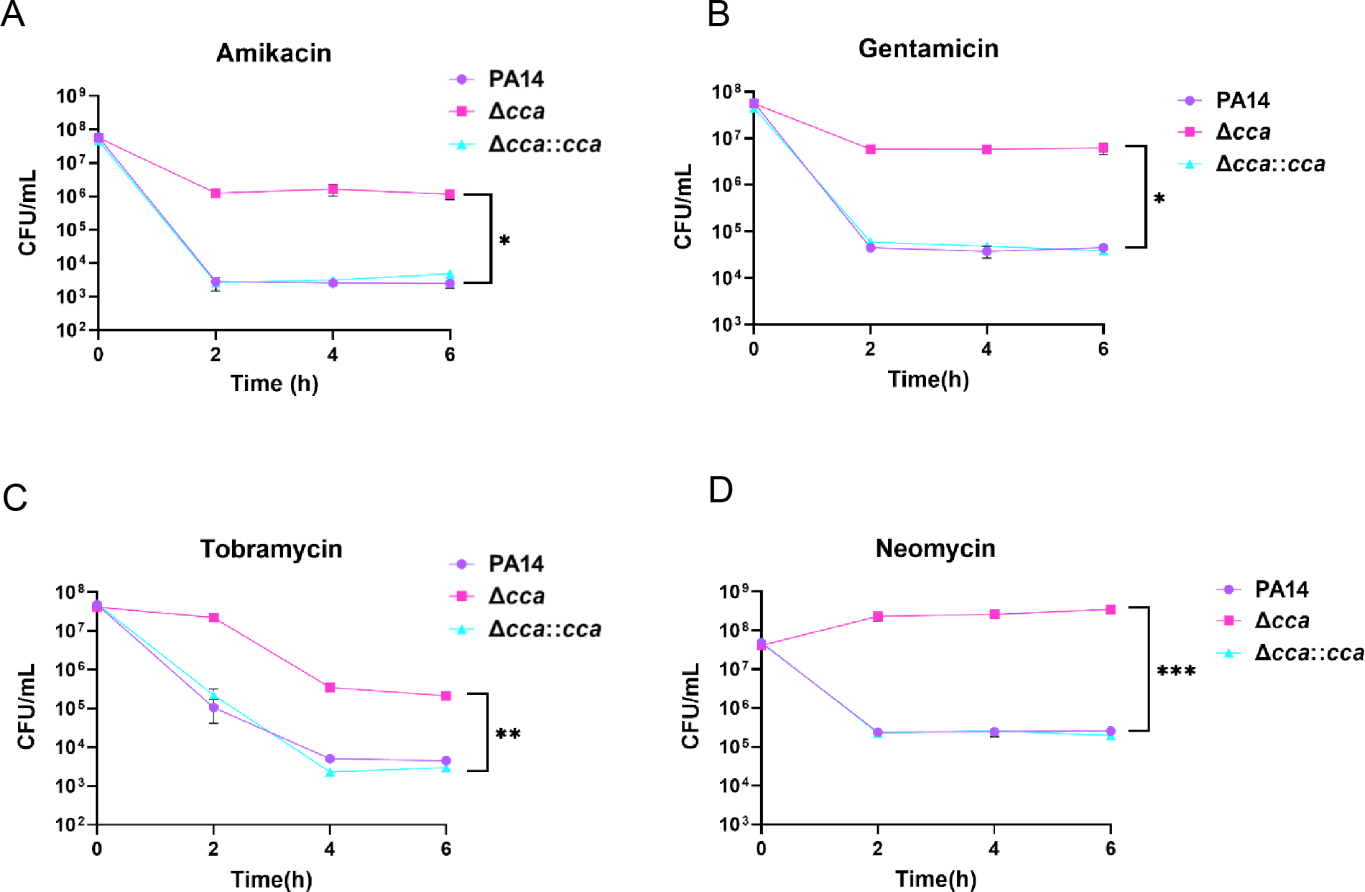
Cca influences tolerance to aminoglycoside antibiotics in *P. aeruginosa*. PA14, the Δ*cca* mutant, and the complemented strain Δ*cca::cca* were grown to an OD_600_ of 1.0 at 37°C and treated with 8 μg/mL amikacin (**A**), 4 μg/mL gentamicin (**B**), 2.4 μg/mL tobramycin (**C**), and 24 μg/mL neomycin (**D**). At the indicated time points, the survival bacterial numbers were determined by serial dilution and plating. *, *P* < 0.05; **, *P* < 0.01; and ***, *P* < 0.001 by Student’s *t*-test.

To assess the role of Cca in antibiotic resistance to aminoglycosides and β-lactams, we examined the resistance of a Δ*cca* mutant to amikacin, gentamicin, tobramycin, neomycin, meropenem, and ceftazidime. Compared with the wild-type strain PA14, the Δ*cca* mutant exhibited the same susceptibility to meropenem and ceftazidime, but decreased susceptibility to amikacin, gentamicin, tobramycin, and neomycin, with twofold increases in minimal inhibitory concentrations (MICs; Table 1). Complementation with a *cca* gene restored susceptibility to these antibiotics (Table 1). Next, we determined the tolerance of PA14 and the Δ*cca* mutant to aminoglycoside antibiotics. As shown in Fig. 1, mutation of the *cca* gene results in approximately 1,000-fold increased survival rates compared to the wild-type PA14 strain following treatment with amikacin, gentamicin, tobramycin, and neomycin, which were reversed by complementation with a *cca* gene. These results indicate a role for Cca in bacterial resistance and tolerance to aminoglycoside antibiotics.

**TABLE 1:** MICs (μg/mL) of antibiotics for the indicated *P. aeruginosa* PA14 strain and its derivatives*^a^*^,^^*b*^

Strain	Amk	Gm	Tob	Neo	Mem	Cef
PA14	2.0	1.0	0.3	3.0	0.25	2.0
Δ*cca*	4.0	2.0	0.6	6.0	0.25	2.0
Δ*cca*::*cca*	2.0	1.0	0.3	3.0	0.25	2.0
Δ*mexXY*	1.0	0.25	0.15	1.5	ND	ND
Δ*cca*Δ*mexXY*	1.0	0.25	0.15	1.5	ND	ND
Δ*armZ*	1.0	0.25	0.15	1.5	ND	ND
Δ*cca*Δ*armZ*	1.0	0.25	0.15	1.5	ND	ND
PA14/pUCP20/pDN19	2.0	1.0	ND	ND	ND	ND
Δ*cca*/pUCP20/pDN19	4.0	2.0	ND	ND	ND	ND
Δ*cca*/pUCP20-*PA4669.1*/pDN19-*PA5149.1*	2.0	1.0	ND	ND	ND	ND
Δ*cca*/pUCP20-*PA5149.1*/pDN19-*PA4669.1*	2.0	1.0	ND	ND	ND	ND

^*a*^ Amk, amikacin; Gm, gentamicin; Tob, tobramycin; Neo, neomycin; Mem, meropenem; Cef, ceftazidime.

^*b*^ ND, not determined.

### Upregulation of *mexXY* contributes to the increased resistance and tolerance to aminoglycosides in the Δ*cca* mutant

**Fig 2:**
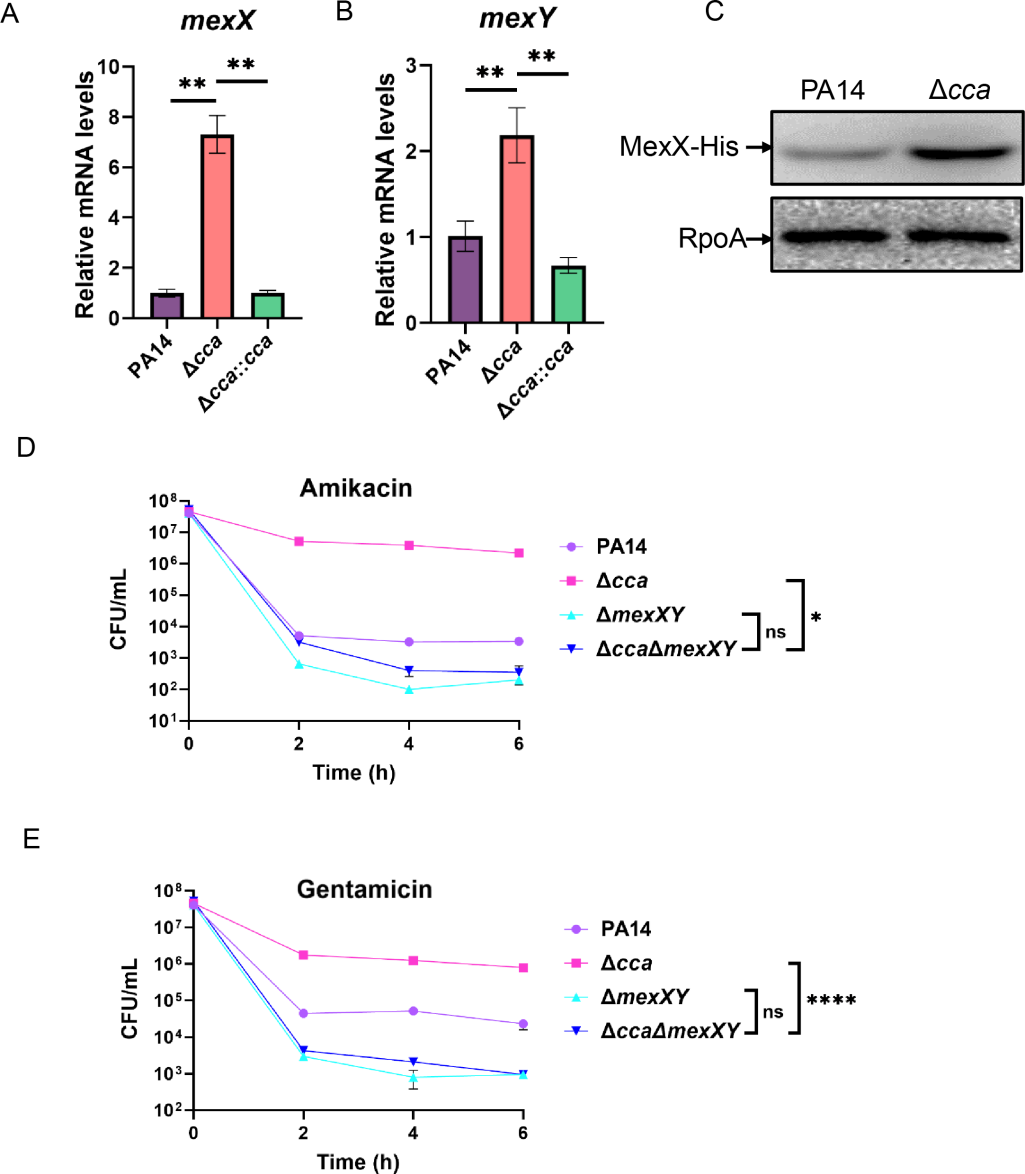
Upregulation of *mexXY* contributes to the increased tolerance to aminoglycoside antibiotics in the Δ*cca* mutant. (**A and B**) Indicated bacterial strains were grown to an OD_600_ of 1.0 at 37°C, followed by RNA purification. Relative mRNA levels of *mexX* (**A**) and *mexY* (**B**) were determined by real-time qPCR with *rpsL* as an internal control. **, *P* < 0.01 by Student’s *t*-test. (**C**) Protein level of MexX-His in PA14 and the Δ*cca* mutant with chromosomally integrated *mexX*-His. The bacterial cells were cultured to an OD_600_ of 1.0 at 37°C, and the MexX-His amounts were examined by western blot with RpoA as the loading control. (**D and E**) PA14, Δ*cca*, Δ*mexXY*, and Δ*cca*Δ*mexXY* were grown to an OD_600_ of 1.0 at 37°C and treated with 8 μg/mL amikacin (**D**) and 4 μg/mL gentamicin (**E**). At the indicated time points, the survival bacterial numbers were determined by serial dilution and plating. ns, not significant, *, *P* < 0.05 and ****, *P* < 0.0001 by Student’s *t*-test.

To understand the mechanism of Cca-mediated regulation of antibiotic susceptibility, we examined the expression levels of the *mexX* and *mexY* genes, which encode the multidrug efflux pump MexXY, a major determinant of aminoglycoside resistance in *P. aeruginosa*. Real-time qPCR results revealed significant upregulation of *mexX* and *mexY* in the Δ*cca* mutant (Fig. 2). Complementation with a *cca* gene restored the relative mRNA levels of *mexX* and *mexY* in the Δ*cca* mutant (Fig. 2). To further confirm the consecutive increase in MexX production due to gene upregulation, we utilized a C-terminal His-tagged *mexX* driven by its native promoter and examined the protein levels of MexX (8). Consistently, the MexX-His protein level was much higher in the Δ*cca* mutant than in PA14 (Fig. 2C). Furthermore, deletion of *mexXY* in the Δ*cca* mutant reduced the MICs and tolerance to the aminoglycosides to the same levels as those in the PA14Δ*mexXY* mutant (Table 1; Fig. 2D and E). These results demonstrate that upregulation of the *mexXY* efflux pump contributes to the increased resistance and tolerance to aminoglycoside antibiotics in the Δ*cca* mutant.

### Upregulation of *armZ* contributes to the increased expression of MexXY and resistance/tolerance to aminoglycosides in the Δ*cca* mutant

**Fig 3:**
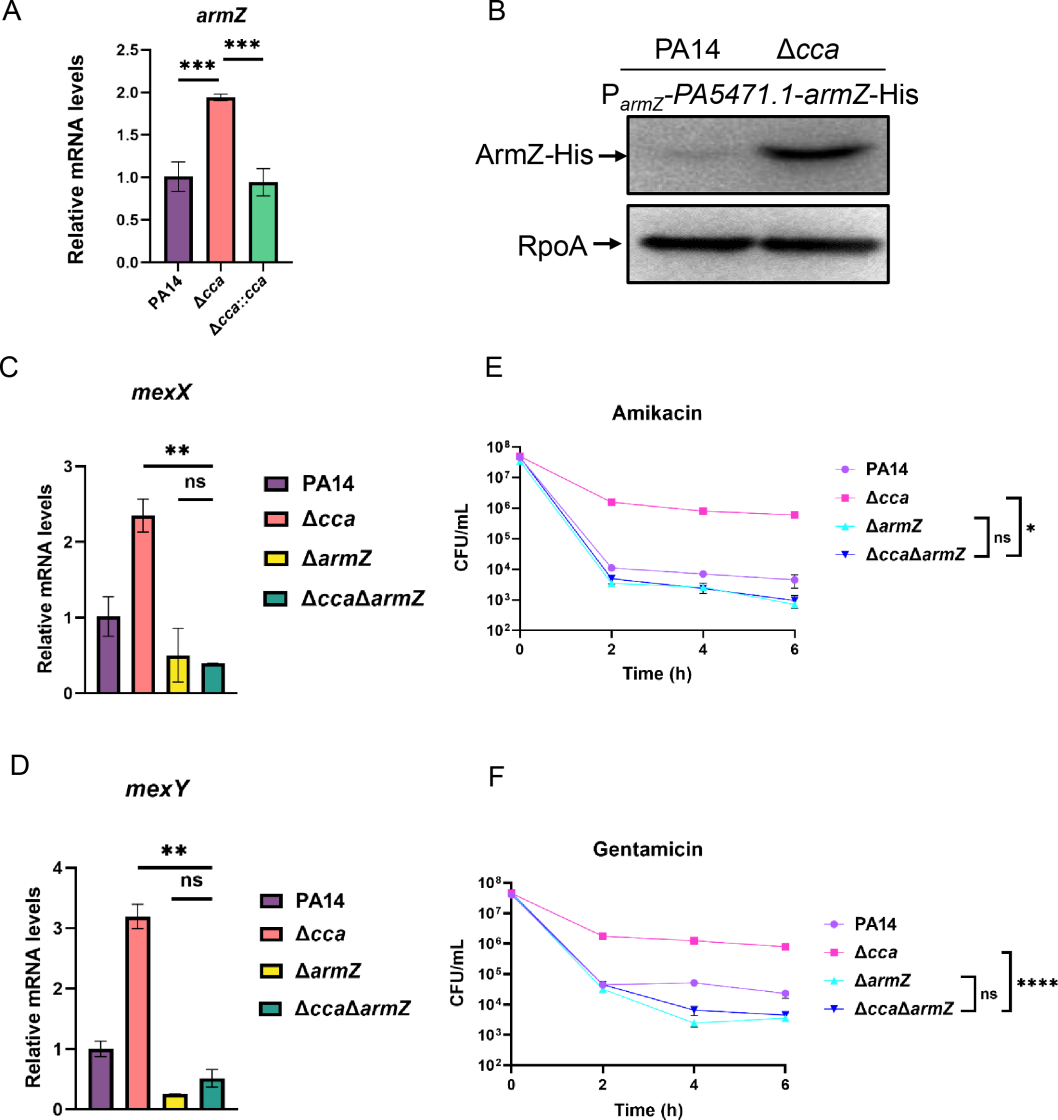
Upregulation of *armZ* contributes to the increased expression of MexXY and tolerance to aminoglycosides in the Δ*cca* mutant. (**A, C, and D**) Indicated bacterial strains were grown to an OD_600_ of 1.0 at 37°C, followed by RNA purification. Relative mRNA levels of *armZ* (**A**) *mexX* (**C**) and *mexY* (**D**) were determined by real-time qPCR with *rpsL* as an internal control. ns, not significant, **, *P* < 0.01 and ***, *P* < 0.001 by Student’s *t*-test. (**B**) Protein level of the ArmZ-His in PA14 and the Δ*cca* mutant containing P*_armZ_-PA5471.1-armZ*-His. The bacterial cells were cultured to an OD_600_ of 1.0 at 37°C, and the ArmZ-His amounts were examined by western blot with RpoA as the loading control. (**E and F**) PA14, Δ*cca*, Δ*armZ*, and Δ*cca*Δ*armZ* were grown to an OD_600_ of 1.0 at 37°C and treated with 8 μg/mL amikacin (**E**) and 4 μg/mL gentamicin (**F**). At the indicated time points, the survival bacterial numbers were determined by serial dilution and plating. ns, not significant, *, *P* < 0.05 and ****, *P* < 0.0001 by Student’s *t*-test.

Transcription of the *mexXY* operon is directly repressed by its repressor MexZ (7). ArmZ controls *mexXY* transcription by interacting with and relieving the repression by MexZ (7). We then examined the transcription levels of *armZ* by real-time qPCR. As shown in Fig. 3A, the relative mRNA level of *armZ* was significantly increased in the Δ*cca* mutant and was restored to the level seen in PA14 when complemented with a *cca* gene. To confirm the upregulation of *armZ*, we utilized a His-tagged *armZ* driven by its native promoter to determine the protein level of ArmZ. Consistent with the increased transcriptional level, ArmZ-His protein was increased in the Δ*cca* mutant (Fig. 3B).

To determine if the upregulated ArmZ contributes to the increased expression of MexXY, we deleted *armZ* in the Δ*cca* mutant and determined the transcription levels of *mexX* and *mexY*. Real-time qPCR revealed that deletion of *armZ* reduced the mRNA levels of *mexX* and *mexY* in the Δ*cca* mutant to the levels seen in the Δ*armZ* mutant (Fig. 3C and D). Consistently, the MICs and tolerance to amikacin and gentamicin of the Δ*cca* mutant were reduced by *armZ* deletion to the same levels seen in the Δ*armZ* mutant of wild-type PA14 (Table 1; Fig. 3E and F). These results suggest that the upregulation of *armZ* contributes to the increased expression of *mexXY* operon and resistance/tolerance to aminoglycosides in the Δ*cca* mutant.

### Cca controls the transcription of *armZ* through its leader peptide PA5471.1

**Fig 4:**
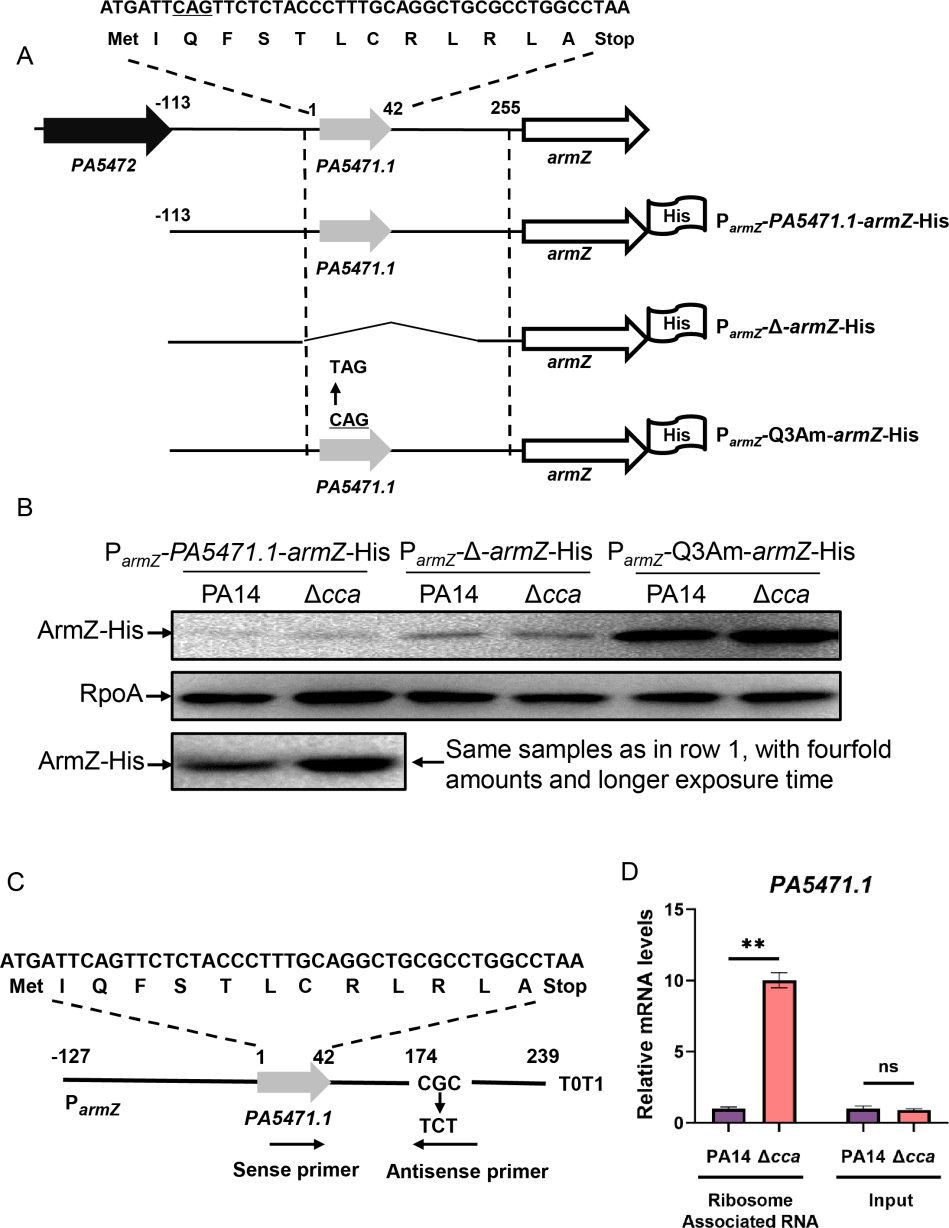
Cca controls the expression of *armZ* through its leader peptide PA5471.1. (**A**) Structures of ArmZ-His fusions. The *armZ*, *PA5471.1*, and upstream regions were highlighted with numbers representing positions relative to the first base pair of the *PA5471.1* start codon as 1. In P*_armZ_*-Δ-*armZ*-His, the fragment from position 1 to 226 was deleted from P*_armZ_-PA5471.1-armZ*-His. In P*_armZ_*-Q3Am-*armZ*-His, a C at position 7 was substituted with a T, generating an amber mutation Q3Am. All the ArmZ-His fusion proteins were constructed in promoterless pUCP20 and transferred into wild-type PA14 and its Δ*cca* mutant. (**B**) ArmZ-His protein amounts from the indicated constructs in PA14 and Δ*cca* were examined by western blot assay. Samples from equivalent bacterial cell numbers were loaded onto sodium dodecyl sulfate-polyacrylamide gel electrophoresis gels and probed with an anti-His or anti-RpoA antibody. Row three shows the same samples as in row 1, but with fourfold amounts and longer exposure time in western blot for easier detection. (**C**) Structure of *PA5471.1*-T0T1. The first base pair of *PA5471.1* start codon was designated as 1. Positions of real-time qPCR primers are indicated by arrows (18). (**D**) Quantification of ribosome-associated *PA5471.1* mRNA. PA14 and the Δ*cca* mutant containing pUCP24-*rplL*-His and pMMB67EH-P*_armZ_-PA5471.1*-T0T1 were lysed by sonication and subjected to Ni-NTA chromatography, followed by RNA extraction. The purified RNA from lysed samples without Ni-NTA chromatography served as Input. The relative mRNA levels of *PA5471.1* were examined by real-time qPCR with 16S ribosomal RNA (*PA0668.1*) as the internal control. ns, not significant, **, *P* < 0.01 by student’s *t*-test.

The expression of *armZ* is regulated through its leader peptide PA5471.1 (10). To explore whether PA5471.1 is involved in the Cca-mediated regulation of *armZ*, we deleted the *PA5471.1* coding region in the ArmZ-His fusion construct, generating P*_armZ_*-Δ-*armZ*-His (Fig. 4A). In another construct, a C was substituted with T at position 7 of *PA5471.1* (encoding a glutamine, Q) to generate an amber mutation (designated P*_armZ_*-Q3Am-*armZ*-His as shown in Fig. 4A), which had previously been shown to promote ribosome stalling and subsequently activate the transcription of *armZ* (10, 18). In agreement with the previous studies (10, 18), deletion or the point mutation of *PA5471.1* largely increased the amounts of ArmZ-His (Fig. 4B). Notably, both mutations led to similar protein amounts of His-tagged ArmZ in wild-type PA14 and its Δ*cca* mutant (Fig. 4B). These data indicate a role for *PA5471.1* in the Cca-mediated regulation of *armZ*.

### Mutation of *cca* enhances ribosome stalling at the *PA5471.1* mRNA

Our previous study demonstrated that a *suhB* mutation increased ribosome stalling at the *PA5471.1* mRNA, leading to increased transcription of *armZ* (18). The involvement of PA*5471.1* in Cca-mediated regulation of *armZ*, as well as its tRNA nucleotidyltransferase function, suggests a role for Cca in modulating ribosome stalling at *PA5471.1* mRNA. Therefore, a modified RNA-binding protein immunoprecipitation assay in combination with real-time qPCR was utilized to examine the amount of ribosome-bound *PA5471.1* mRNA as previously described (18–20). Plasmids pMMB67EH-P*_PA5471 (armZ)_-PA5471.1*-T0T1 and pUCP24-*rplL*-His, previously constructed and utilized for the modified RNA-binding protein immunoprecipitation assay (18), were introduced into PA14 and its Δ*cca* strains. In the plasmid pMMB67EH-P*_armZ_-PA5471.1*-T0T1, two nucleotide substitutions were introduced into the *PA5471.1* downstream region (Fig. 4C), ensuring that RNA transcribed from the chromosome would not be detected by real-time qPCR primers designed for the substituted region. Ribosomes from the two bacterial strains were isolated using Ni-NTA chromatography, and the associated RNA was purified and subjected to real-time qPCR. The mRNA levels of *PA5471.1* produced from pMMB67EH-P*_armZ_-PA5471.1*-T0T1 were similar between PA14 and the Δ*cca* mutant (Input in Fig. 4D). However, a significantly increased amount of *PA5471.1* mRNA was associated with ribosomes isolated from the Δ*cca* mutant (Fig. 4D), indicating enhanced ribosome stalling at the *PA5471.1* mRNA.

### 3Q4F of PA5471.1 is involved in the Cca-mediated resistance/tolerance to aminoglycosides

**Fig 5:**
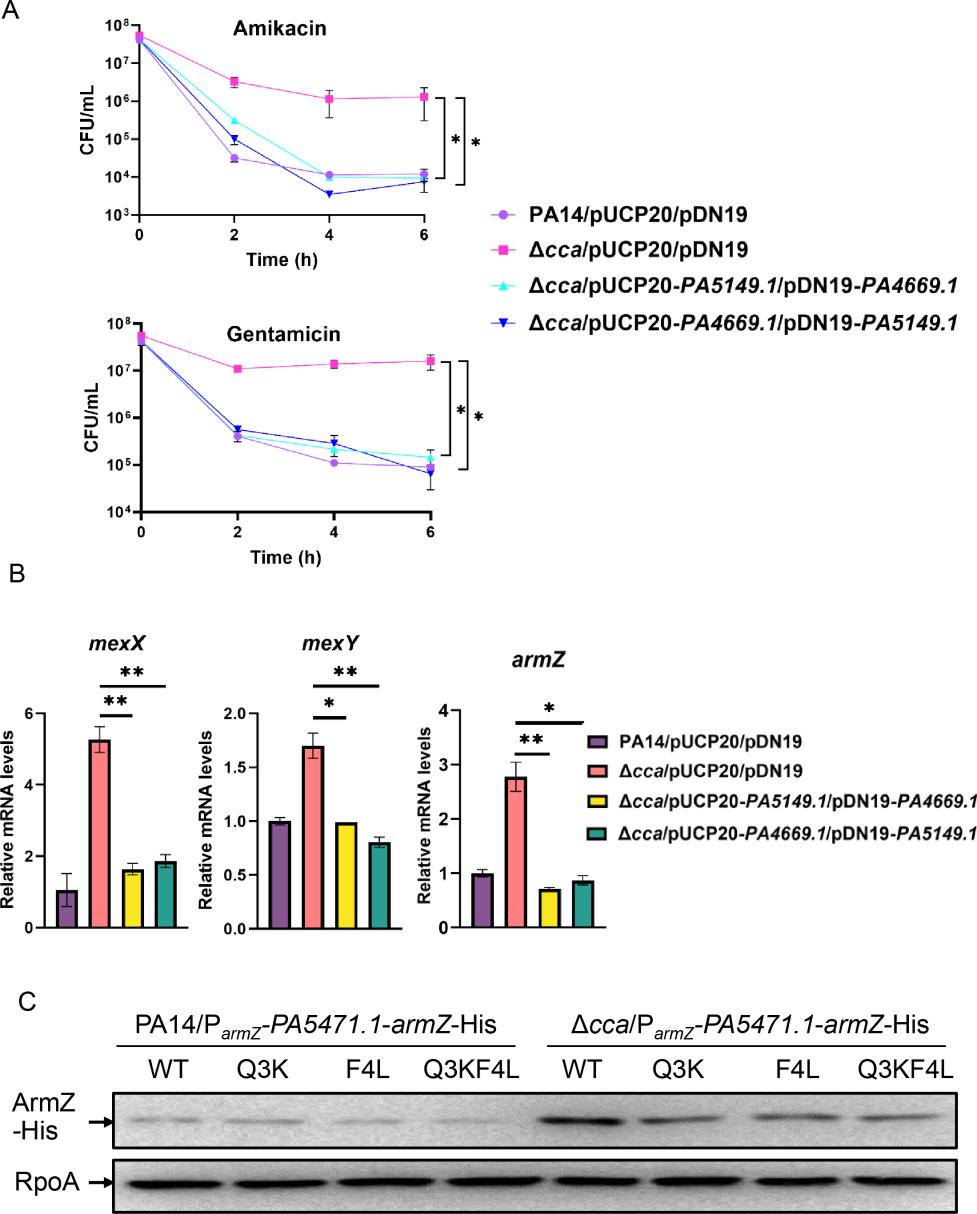
Glutamine at the third and phenylalanine at the fourth position of PA5471.1 are involved in the Cca-mediated tolerance to aminoglycosides. (**A**) Indicated bacterial cells were grown to an OD_600_ of 1.0 at 37°C and treated with 8 μg/mL amikacin or 4 μg/mL gentamicin. At the indicated time points, the survival bacterial numbers were determined by serial dilution and plating. *, *P* < 0.05 by Student’s *t*-test. (**B**) Indicated bacterial strains were grown to an OD_600_ of 1.0 at 37°C, followed by RNA purification. Relative mRNA levels of *mexX*, *mexY,* and *armZ* were determined by real-time qPCR with *rpsL* as an internal control. *, *P* < 0.05 and **, *P* < 0.01 by Student’s *t*-test. (**C**) Protein level of the ArmZ-His in PA14 and its Δ*cca* mutant. Indicated bacterial cells were cultured to an OD_600_ of 1.0 at 37°C, and the ArmZ-His amounts were examined by western blot with RpoA as the loading control.

Ribosome stalling at the leader peptide PA5471.1 resulted in an increase in ArmZ levels in the Δ*cca* mutant compared to those of PA14. As a tRNA nucleotidyltransferase, Cca is responsible for adding the CCA triplet at the 3′ termini of tRNAs to facilitate aminoacylation (21). It is possible that in the absence of Cca, some specific tRNAs lacking the 3′ CCA triplet have reduced or no aminoacylation, resulting in ribosome stalling at *PA5471.1*, increased *armZ* expression, and consequently increased resistance and tolerance to aminoglycosides. Therefore, we analyzed the tRNA coding genes in *P. aeruginosa* (www.pseudomonas.com). We found 63 tRNA encoding genes (Table S1), among which tRNA-Gln (glutamine) and tRNA-Phe (phenylalanine) were each encoded by only one gene, namely *PA4669.1* and *PA5149.1*, respectively, which have only the CC sequence at their 3′ terminal ends. Glutamine is located at the third, while phenylalanine is located at the fourth position of the PA5471.1. To investigate if *PA4669.1* and *PA5149.1* are involved in the Cca-mediated regulation of resistance and tolerance to aminoglycosides, we overexpressed 3′ terminal A-added *PA4669.1* and *PA5149.1* in the Δ*cca* mutant. Individual expression of the two modified tRNAs had no influence on the tolerance to aminoglycosides (Fig. S1). However, simultaneous expression of them (in two different plasmids, pUCP20 and pDN19) restored the MICs and tolerance to amikacin and gentamicin of the Δ*cca* mutant to that of the wild-type PA14 strain (Table 1; Fig. 5A). In addition, simultaneous expression of the two modified tRNAs decreased the expression of *mexX*, *mexY*, and *armZ* (Fig. 5B).

To further confirm that glutamine at the third and phenylalanine at the fourth (3Q4F) position of the PA5471.1 plays a role in the Cca-mediated regulation of ArmZ, we introduced a C to A point mutation at the 7th position (Q3K), a C to A point mutation at the 12th position (F4L), as well as simultaneous C to A at the 7th and C to A at the 12th position mutations (Q3KF4L) in *PA5471.1* of the P*_armZ_-PA5471.1-armZ*-His construct, resulting in Q3K, F4L, and Q3KF4L amino acid substitutions. Western blot assay was performed to examine the production of ArmZ. Consistent with a previous report that the Q3K mutation did not enhance *armZ* expression (10), individual Q3K and F4L substitutions did not increase the expression of ArmZ in the wild-type PA14 strain, suggesting that these two substitutions did not result in ribosome stalling at *PA5471.1* (Fig. 5C). Importantly, the Q3K, F4L, and Q3KF4L simultaneous mutation decreased the expression of ArmZ in the Δ*cca* mutant, while having no obvious effect in the wild-type PA14 strain (Fig. 5C). These results suggest that Q3 and F4 are involved in the Cca-mediated regulation of resistance and tolerance to aminoglycosides in *P. aeruginosa*.

## DISCUSSION

**Fig 6:**
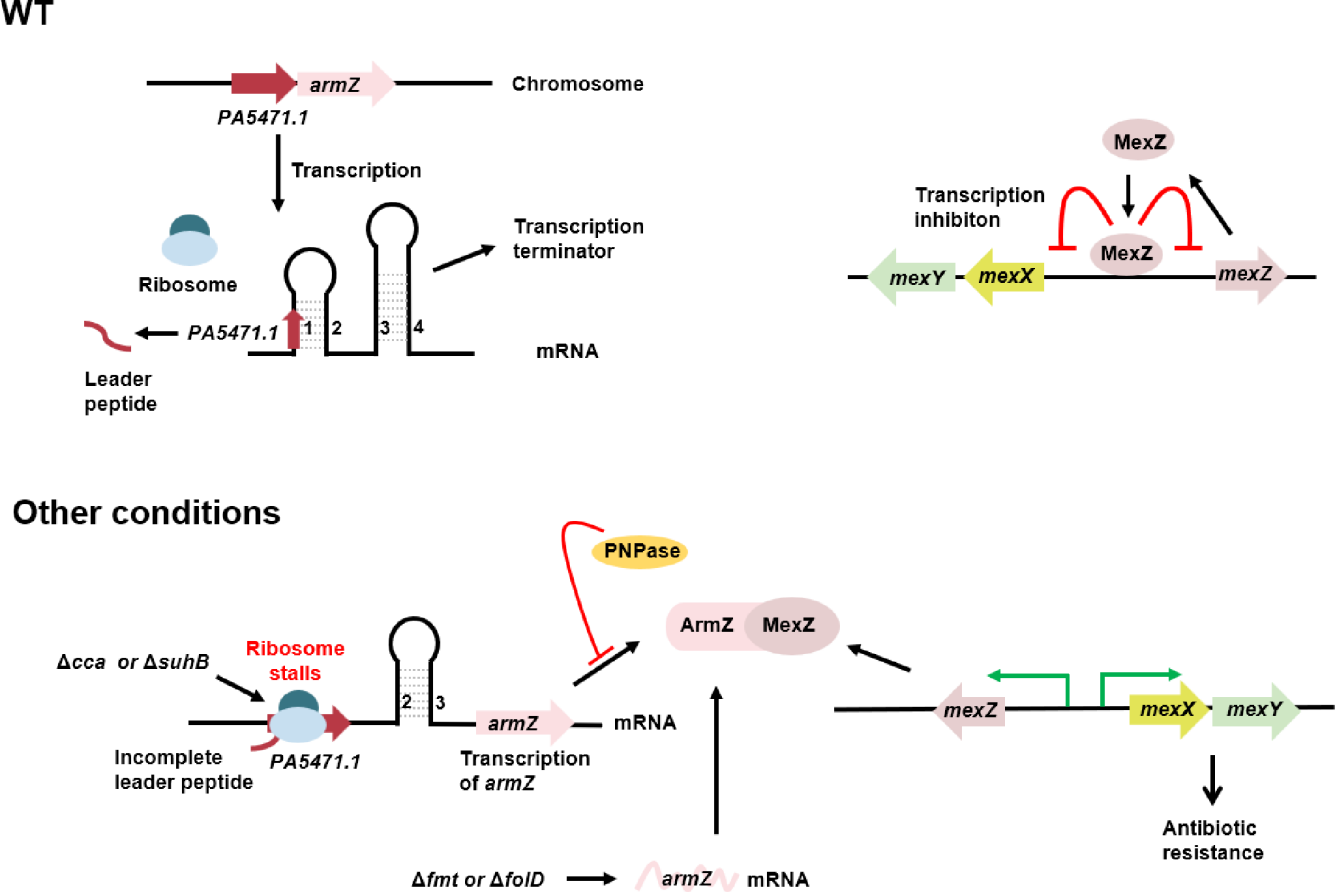
Proposed model of *mexXY* regulation mediated by MexZ, ArmZ, SuhB, PNPase, Cca, Fmt, and FolD in *P. aeruginosa* based on this and previous studies (7, 8, 10, 18, 23–25). The *armZ* is regulated via a transcription attenuation mechanism. *armZ* and its leader peptide (*PA5471.1*) are transcribed under a constitutive promoter. In the wild-type strain, complete translation of *PA5471.1* results in the formation of a transcription terminator (the hairpin structure formed by the base pairing between segments 3 and 4), which blocks the transcription of downstream *armZ* (10). Meanwhile, MexZ binds to the intergenic region and inhibits transcription of *mexXY* and *mexZ* (24). In the absence of *suhB* or *cca*, ribosome stalls at the *PA5471.1* mRNA, which alters the RNA secondary structure and abolishes the formation of the transcription terminator, resulting in *armZ* transcription (18). PNPase represses translation of the *armZ* (8). The mutation of *fmt* and *folD* also leads to upregulation of *armZ* (25). The ArmZ protein binds to MexZ and releases its repression on its own gene and *mexXY* (7, 23).

The MexXY multidrug efflux pump is an important contributor to aminoglycoside resistance and tolerance in both *P. aeruginosa* reference strains and clinical isolates (22). Several regulators of MexXY have been found in *P. aeruginosa*, including regulators MexZ, ArmZ, SuhB, PNPase, Fmt, and FolD (Fig. 6) (7, 8, 10, 18, 23–25). In this study, we showed that tRNA nucleotidyltransferase Cca also controls the expression of MexXY in *P. aeruginosa*. Similar to SuhB and PNPase, Cca regulates the expression of MexXY by modulating the expression of ArmZ (Fig. 6).

tRNAs are initially transcribed as precursor tRNAs that undergo extensive processing at both the 5′ and 3′ ends to generate functional tRNAs that can be charged with their cognate amino acids at the 3′-ends (26). At the 3′-end, each functional tRNA includes the universally invariant CCA triplet that is either encoded in the tRNA coding gene or posttranscriptionally added by a highly specialized RNA polymerase, tRNA nucleotidyltransferase (CCA-adding enzyme) (26). In some bacteria, such as *E. coli*, *Vibrio cholerae*, and *Yersinia pestis*, the CCA 3′ sequence is already encoded in all tRNA genes (14, 27). Therefore, disruption of the *cca* gene in *E. coli* is not lethal, resulting only in a decreased growth rate (14). In the present study, we found that deletion of *cca* did not affect bacterial survival but led to a reduced growth rate in *P. aeruginosa* (Fig. S2). However, based on a previous study and the analysis of all the tRNA genes on the *Pseudomonas* website (www.pseudomonas.com) (27), not all cognate tRNAs for amino acids encode the 3′ CCA terminus. For example, *PA5149.1*, the only tRNA-Phe encoding gene, lacks the 3′ CCA sequence. Therefore, it is possible that other 3′ CCA-adding enzymes or other modification mechanisms exist for tRNA 3′ CCA modification and repair in *P. aeruginosa*.

Of note, besides their canonical role in mRNA translation, tRNAs have also been reported to perform additional functions in the regulation of gene expression (28). Uncharged tRNAs have been demonstrated to play a role in the bacterial stringent response (28, 29). Uncharged tRNAs function as effector molecules to regulate the expression of genes involved in aminoacyl-tRNA synthetases, amino acid biosynthesis, and amino acid uptake in *Bacillus subtilis* and other gram-positive bacteria (30). In addition, recent studies have revealed that tRNA modifications play important roles in regulating bacterial pathogenicity, virulence factors, and oxidative stress response (31–34). To our knowledge, this study provides the first evidence that tRNA nucleotidyltransferase Cca controls susceptibility to aminoglycosides through the MexXY efflux pump in *P. aeruginosa*. However, its global regulatory role in *P. aeruginosa* is still unknown and needs further investigation.

Interestingly, in some bacterial species, such as *Bacillus halodurans*, *Deinococcus radiodurans*, and *Aquifex aeolicus*, the incorporation of C and A residues is split between two tRNA nucleotidyltransferases (35–37). One CC-adding enzyme adds two C residues to the 3′-end of the tRNA, and the other A-adding enzyme incorporates the terminal A, whose collaboration leads to the complete CCA-end (36, 37). According to website prediction (www.pseudomonas.com) and a previous study, only one tRNA nucleotidyltransferase was found in *P. aeruginosa* (35). Therefore, it is possible that Cca acts as a tRNA nucleotidyltransferase with dual specificity for adding both C and A in *P. aeruginosa*.

Growth rate also influences bacterial susceptibility to antibiotics (38). The increased resistance and tolerance to aminoglycosides might be due to the decreased growth rate of the Δ*cca* mutant. However, deletion of the *mexXY* efflux pump genes maintained the reduced growth rate (data not shown) while restoring the resistance and tolerance to aminoglycoside antibiotics of the Δ*cca* mutant. Therefore, the decreased susceptibility to aminoglycosides of the Δ*cca* mutant is not due to the reduced growth rate. In addition, simultaneous expression of the 3′ A-added *PA4669.1* and *PA5149.1* restored susceptibility to aminoglycosides but not the growth rate of the Δ*cca* mutant (data not shown). The inability to complement the growth rate indicates that additional functions, other than A-addition to *PA4669.1* and *PA5149.1*, affect bacterial growth in *P. aeruginosa*.

In summary, we found that tRNA nucleotidyltransferase Cca controls susceptibility to aminoglycoside antibiotics in *P. aeruginosa* and revealed its regulatory role on *armZ* expression and thus the *mexXY* efflux pump.

## MATERIALS AND METHODS

### Bacterial strains, plasmids, and primers

The bacterial strains, plasmids, and primers used in this study are listed in Tables S2 and S3. Bacterial strains *P. aeruginosa* (PA14) and *E. coli* used in this study were cultured in lysogeny broth (LB) medium (containing 1% NaCl, 1% tryptone, and 0.5% yeast extract; all [wt/vol]) or on LB agar plates (supplemented with 1.5% agar) at 37°C. For maintenance of plasmids within bacteria, appropriate antibiotics were supplemented in the medium at the following final concentrations: for *P. aeruginosa*, 50 μg/mL gentamicin, 150 μg/mL carbenicillin, and 50 μg/mL tetracycline; for *E. coli*, 10 μg/mL gentamicin, 100 μg/mL ampicillin, and 10 μg/mL tetracycline.

### Construction of plasmids and deletion mutants

The *cca* gene was knocked out in frame by homologous recombination as previously documented (39). Upstream and downstream fragments (approximately 1,000 bp) of the *cca* gene were amplified by PCR with PA14 genomic DNA as a template and primers shown in Table S3. The PCR products were cloned into the *Eco*RI*-Xba*I sites of pEX18Tc plasmid. Then the deletion construct pEX18Tc-*cca* was transferred into *E. coli* S17-1 by electroporation, followed by conjugal transfer into PA14. After that, single-crossover strains were selected on plates containing 50 μg/mL tetracycline and 25 μg/mL kanamycin. An overnight culture of the single-crossover strain was plated onto LB agar plates containing 7.5% sucrose to select double-crossover strains. Deletion of the *cca* gene was confirmed by PCR. Deletion of *mexXY* or *armZ* in PA14 or Δ*cca* was carried out with a similar procedure using the plasmid pEX18Tc-*mexXY* or pEX18Tc-*armZ* constructed in a previous study (40).

To complement the *cca* gene, the *cca* gene with its native promoter was amplified by PCR using specific primers (Table S3) with PA14 genomic DNA as the template. The PCR product was digested with *Eco*RI-*Bam*HI and then cloned into pUC18T-mini-Tn7T (41). Then the resultant plasmid was introduced into the chromosome of the Δ*cca* mutant by electroporation along with the helper plasmid pTNS3 (42). Insertion of the *cca* gene into the chromosome was verified by PCR amplification with primers P*_glmS_*_-down_ and P*_Tn7R_* (Table S3).

To construct plasmid pUCP20-*PA5149.1*, the fragment of *PA5149.1* with a 3′ adenine addition was amplified by PCR using specific primers (Table S3) with PA14 genomic DNA as a template. The PCR products were digested with *Eco*RI*-Bam*HI and then cloned into pUCP20 plasmid. pUCP20-*PA4669.1*, pDN19-*PA5149.1*, and pDN19-*PA4669.1* were constructed using a similar strategy.

To generate the construct of P*_armZ_-PA5471.1-armZ*-His, the C-terminal His-tagged *armZ* with its native promoter region was PCR amplified with PA14 genomic DNA as a template and inserted into *Sac*I*-Bam*HI sites of the promoterless pUCP20 (43, 44). To construct P*_armZ_*-Δ-*armZ*-His, the 113 bp DNA fragment upstream of *PA5471.1* and the C-terminal His-tagged *armZ* gene with its SD sequence were amplified using specific primers and P*_armZ_-PA5471.1-armZ*-His as a template (Table S3). The fragments were ligated by overlapping PCR and subsequently cloned into the *Sac*I*-Bam*HI site of the promoterless pUCP20. For P*_armZ_*-Q3Am-*armZ*-His, a point mutation (leading to the substitution of a glutamine to an amber stop codon, Q3Am) was introduced into primers P*_armZ_*-Q3Am-*armZ*-UR and P*_armZ_*-Q3Am-*armZ*-DF. Two DNA fragments were PCR amplified with P*_armZ_*-Q3Am-*armZ*-UF/P*_armZ_*-Q3Am-*armZ*-UR and P*_armZ_*-Q3Am-*armZ*-DF/P*_armZ_*-Q3Am-*armZ*-DR, ligated by overlapping and then cloned into *Sac*I*-Bam*HI site of the promoterless pUCP20. P*_armZ_-PA5471.1*_Q3K_-*armZ*-His, P*_armZ_-PA5471.1*_F4L_-*armZ*-His, and P*_armZ_-PA5471.1*_Q3KF4L_-*armZ*-His were constructed with similar procedures.

### MIC determination

*P. aeruginosa* strains were subcultured in LB to an OD_600_ of 1.0 (5 × 10^8^ CFU/mL). MICs were determined with an initial inoculum 1 × 10^5^ bacterial cells each well in Cation-adjusted Mueller–Hinton Broth (Ca-MHB, QDRS Biotec, Qingdao, China) using a twofold dilution method in accordance with the guidelines of the Clinical and Laboratory Standards Institute (45). MICs were defined as the lowest concentration of antibiotic that inhibits visible growth following 24 h incubation at 37°C. Each experiment was repeated three times.

### Bacterial survival assay

Bacterial cells were subcultured to an OD_600_ of 1.0 and 10-fold diluted into 2 mL of fresh LB medium. Then bacterial cells were treated with aminoglycoside antibiotics at final concentrations of twofold MIC (amikacin and gentamicin) or fourfold MIC (tobramycin and neomycin) at 37°C with shaking at 200 rpm. At indicated intervals (0, 2, 4, and 6 h), viable bacterial numbers were determined by serial dilution and plating assay. All experiments were performed in triplicate.

### RNA isolation and real-time qPCR

Overnight bacterial cultures were 50-fold diluted into fresh LB medium and grown to an OD_600_ of 1.0. Total RNA was isolated utilizing the Bacterial Total RNA Isolation Kit (Zomanbia, Beijing, China), and cDNA was synthesized using a reverse transcriptase (Vazyme, Nanjing, China) and random primers (Vazyme, Nanjing, China). Real-time qPCR was performed using the ChamQ Universal SYBR qPCR Master Mix (TransGen Biotech, Beijing, China) and specific forward and reverse qPCR primers (Table S3) in a CFX Connect Real-Time system (Bio-Rad, United States). *rpsL*, the 30S ribosomal protein S12 encoding gene, was used as an internal control (46).

### Western blot assay

Overnight bacterial culture was 50-fold diluted into fresh LB medium and grown to an OD_600_ of 1.0. Then samples from equivalent numbers of bacterial cells were collected by centrifugation, resuspended in 1× SDS loading buffer (62.5 mM Tris-HCl [pH 6.8], 10% [vol/vol] glycerol, 2% [wt/vol] SDS, 1% [vol/vol] β-mercaptoethanol, and 0.02% [wt/vol] bromophenol blue), boiled at 99°C for 10 min, and then separated by 12% sodium dodecyl sulfate-polyacrylamide gel electrophoresis gels. Proteins were transferred onto a polyvinylidene difluoride membrane and probed with a mouse monoclonal His antibody or RNA polymerase α antibody (RpoA, Biolegend). The signals were detected using the Immobilon Western Chemiluminescent HRP Substrate kit (Millipore). RpoA here serves as a loading control.

### Detection of *PA5471.1* mRNA associated with ribosomes

The amount of *PA5471.1* mRNA associated with ribosomes was determined using a modified RNA-binding protein immunoprecipitation method followed by real-time qPCR as previously described (18–20). pMMB67EH-P*_armZ_-PA5471.1*-T0T1 and pUCP24-*rplL*-His, previously utilized in the RNA-binding protein immunoprecipitation (18), were introduced into PA14 and its Δ*cca* mutant. Bacterial cells were grown to an OD_600_ of 1.0 and collected by centrifugation. Ribosomes were purified by Ni-affinity chromatography as in a previous study with modifications (47). In brief, the collected bacterial cells were suspended in lysis buffer (150 mM NaCl, 20 mM Tris-HCl, 10 mM imidazole, 3 mM β-mercaptoethanol, 0.5% NP-40, pH 8.0) and lysed by sonication. Supernatants were collected after centrifugation and incubated with Ni-NTA agarose beads at 4°C for 1 h. The beads were washed four times with lysis buffer, and then the ribosomes were eluted using elution buffer, followed by RNA purification with an RNA Prep Pure Cell/Bacteria Kit (TransGen Biotech, Beijing, China). The amount of *PA5471.1* mRNA was determined by real-time qPCR with specific primers (Table S3) using the 16S ribosomal RNA encoding gene *PA0668.1* as the internal control for normalization.

### Statistical analysis

Statistical analyses were conducted using GraphPad Prism 9.0 software. Two-tailed unpaired Student’s *t*-tests were employed to calculate *P* values, with statistical significance set at *P* < 0.05.

## SUPPLEMENTAL MATERIAL

Fig. S1 and S2 aac.01653-25-s0001.pdf Bacterial tolerance and growth curve.

Table S1 aac.01653-25-s0002.xlsx tRNAs in *P. aeruginosa*.

Table S2 aac.01653-25-s0003.docx Strains and plasmids used in this study.

Table S3 aac.01653-25-s0004.doc Primers used in this study.

ASM does not own the copyrights to Supplemental Material that may be linked to, or accessed through, an article. The authors have granted ASM a non-exclusive, world-wide license to publish the Supplemental Material files. Please contact the corresponding author directly for reuse.
