# Supplementary material for: Pseudomonas aeruginosa tRNA nucleotidyltransferase Cca controls resistance and tolerance to aminoglycoside antibiotics by regulating the MexXY multidrug efflux pump
Source: Antimicrob Agents Chemother. 2026 Mar 3;70(4):e01653-25. doi: 10.1128/aac.01653-25 (PMC13041342; doi:10.1128/aac.01653-25)
Supplement: Fig. S1 and S2 — Bacterial tolerance and growth curve. [file aac.01653-25-s0001.pdf]

Fig. S1

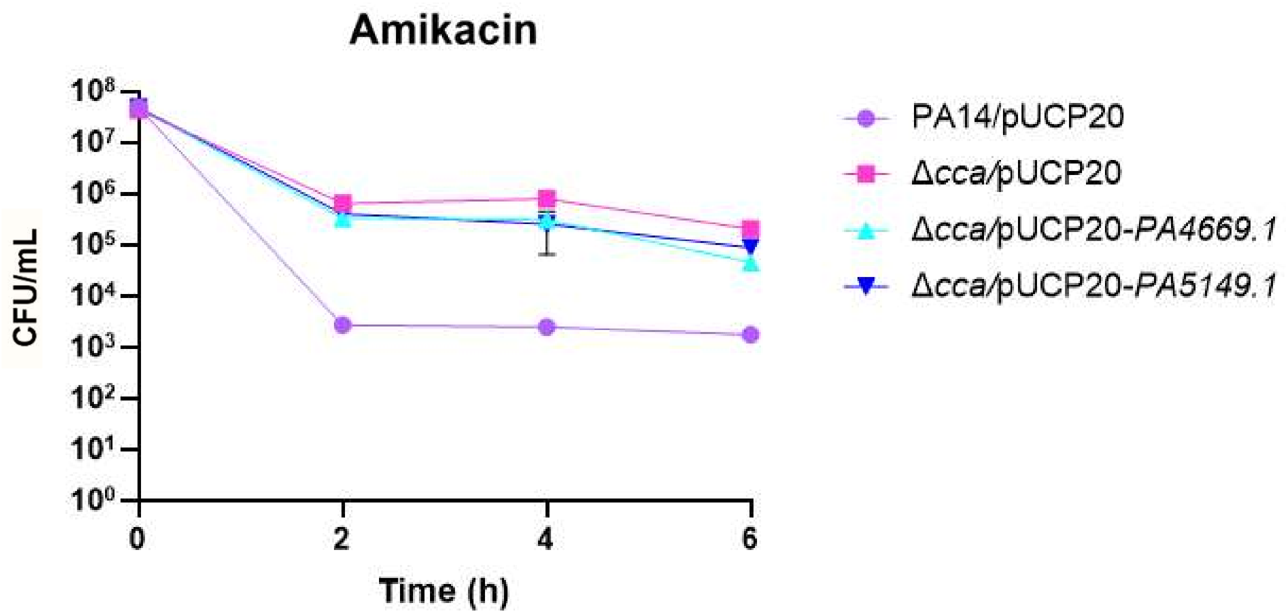

Fig. S1. Bacterial tolerance to amikacin. Individual expression of 3' terminal A-added *PA4669.1* or *PA5149.1* can not restore bacterial tolerance to amikacin in the Δ*cca* mutant. Indicated bacterial cells were grown to an OD_{600} of 1.0 at 37 °C and treated with 8 µg/mL amikacin. At indicated time points, the survival bacterial numbers were determined by serial dilution and plating.

Fig. S2

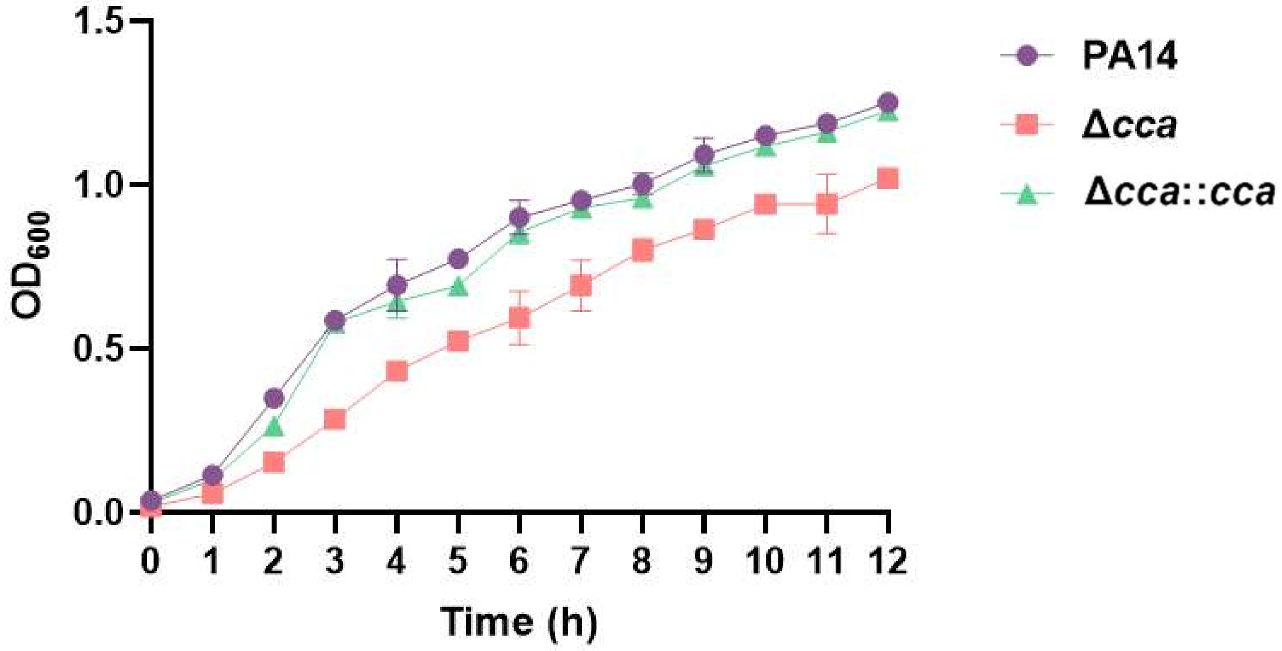

Fig. S2. Growth curves of PA14, the Δ*cca* mutant and the complemented strain Δ*cca*::*cca*.
