# Supplementary material for: Pseudomonas aeruginosa tRNA nucleotidyltransferase Cca controls resistance and tolerance to aminoglycoside antibiotics by regulating the MexXY multidrug efflux pump
Source: Antimicrob Agents Chemother. 2026 Mar 3;70(4):e01653-25. doi: 10.1128/aac.01653-25 (PMC13041342; doi:10.1128/aac.01653-25)
Supplement: Table S2 — Strains and plasmids used in this study. [file aac.01653-25-s0003.docx]

**Table S2.** *E. coli*, *P. aeruginosa* strains (PA14 and its derivatives) and plasmids used in this study.

| Strains or plasmids | Description | Source or reference |
| --- | --- | --- |
| **strains** |  |  |
| *E. coli* strain |  |  |
| DH5α | F^̶^ ϕ 80d*lacZ*∆M15 *endA1 recA1 hsdR17*(r_K_^̶^ m_K_^+^) *supE44 thi-1 relA1* ∆(*lacZYA-argF*)*U169 gyrA96 deoR* | TransGene |
| S17-1 | RP4-2 Tc::Mu Km::Tn*7* Tp^r^ Sm^r^ Pro Res^̶^ Mod^+^ | Stratagene |
| *P. aeruginosa strains* |  |  |
| PA14 | Wild-type strain PA14 | [1] |
| Δ*cca* | PA14 with an in-frame deletion of the *cca* gene | This study |
| Δ*cca*::*cca* | Δ*cca* complementation with *cca* inserted on chromosome | This study |
| Δ*mexXY* | PA14 with deletion of the *mexXY* operon | This study |
| Δ*cca*Δ*mexXY* | PA14 with deletion of the *mexXY* operon and an in-frame deletion of the *cca* gene | This study |
| Δ*armZ* | PA14 with deletion of the *armZ* | This study |
| Δ*cca*Δ*armZ* | PA14 with deletion of the *armZ* and an in-frame deletion of the *cca* gene | This study |
| **Plasmids** |  |  |
| pUCP20 | shuttle vector between *E. coli* and *P. aeruginosa*; Amp^r^ | [2] |
| pUCP20-*PA5149.1* | *PA5149.1* gene with a 3’ terminal adenine addition from PA14 in pUCP20; Amp^r^ | This study |
| pUCP20-*PA4669.1* | *PA4669.1* gene with a 3’ terminal adenine addition from PA14 in pUCP20; Amp^r^ | This study |
| P*_armZ_*-*PA5471.1*-*armZ*-His | His-tagged *armZ* gene with its native promoter in promoterless pUCP20; Amp^r^ | This study |
| P*_armZ_*-Δ-*armZ*-His | P*_armZ_*-*PA5471.1*-*armZ*-His with *PA5471.1* deletion; Amp^r^ | This study |
| P*_armZ_*-Q3Am-*armZ*-His | P*_armZ_*-*PA5471.1*-*armZ*-His with Q3Am substitution in *PA5471.1*; Amp^r^ | This study |
| P*_armZ_*-*PA5471.1*_Q3K_-*armZ*-His | P*_armZ_*-*PA5471.1*-*armZ*-His with Q3K substitution in *PA5471.1*; Amp^r^ | This study |
| P*_armZ_*-*PA5471.1*_F4L_-*armZ*-His | P*_armZ_*-*PA5471.1*-*armZ*-His with F4L substitution in *PA5471.1*; Amp^r^ | This study |
| P*_armZ_*-*PA5471.1*_Q3KF4L_-*armZ*-His | P*_armZ_*-*PA5471.1*-*armZ*-His with Q3K and F4L substitutions in *PA5471.1*; Amp^r^ | This study |
| pDN19 | shuttle vector between *E. coli* and *P. aeruginosa*; Tc^r^ | [3] |
| pDN19-*PA5149.1* | *PA5149.1* gene with a 3’ terminal adenine addition from PA14 in pDN19; Tc^r^ | This study |
| pDN19-*PA4669.1* | *PA4669.1* gene with a 3’ terminal adenine addition from PA14 in pDN19; Tc^r^ | This study |
| pUC18T-mini-Tn7T-Tc | mini-Tn7 base vector insertion into chromosome attTn7 site, Tc^r^ | [4] |
| pUC18T-mini-Tn7T-*cca* | *cca* gene with its native promoter from PA14 in pUC18T-mini-Tn7T; Tc^r^ | This study |
| pUC18T-mini-Tn7T-*mexX*-His | His-tagged *mexX* gene with its native promoter in pUC18T-mini-Tn7T; Tc^r^ | [5] |
| pEX18Tc | gene knockout vector; Tc^r^ | [6] |
| pEX18Tc-*cca* | *cca* gene in-frame deletion on pEX18Tc; Tc^r^ | This study |
| pEX18Tc-*mexXY* | *mexXY* operon deletion on pEX18Tc; Tc^r^ | [7] |
| pEX18Tc-*armZ* | *armZ* gene deletion on pEX18Tc; Tc^r^ | [7] |
| pUCP24-*rplL*-His | His-tagged ribosomal protein RplL in pUCP24; Gm^r^ | [8] |
| pMMB67EH-P*_PA5471_*-*PA5471.1*-T0T1 | *PA5471.1* with the promoter of *PA5471* followed by two transcriptional terminators in promoterless pMMB67EH; Amp^r^ | [8] |

1. Liberati, N.T., et al., An ordered, nonredundant library of *Pseudomonas aeruginosa* strain PA14 transposon insertion mutants*.* Proc Natl Acad Sci U S A, 2006. **103**(8): p. 2833-2838.
2. West, S.E., et al., Construction of improved *Escherichia-Pseudomonas* shuttle vectors derived from pUC18/19 and sequence of the region required for their replication in *Pseudomonas aeruginosa.* Gene, 1994. **148**(1): p. 81-86.
3. Li, K., et al., SuhB is a regulator of multiple virulence genes and essential for pathogenesis of *Pseudomonas aeruginosa.* mBio, 2013. **4**(6): p. e00419-13.
4. Choi, K.H., et al., mini-Tn7 insertion in bacteria with single attTn7 sites: example *Pseudomonas aeruginosa.* Nat Protoc, 2006. **1**: p. 153-61.
5. Fan Z., et al., *Pseudomonas aeruginosa* Polynucleotide Phosphorylase Controls Tolerance to Aminoglycoside Antibiotics by Regulating the MexXY Multidrug Efflux Pump. Antimicrob Agents Chemother, 2021. **65**(2).
6. Hoang, T.T., et al., A broad-host-range Flp-FRT recombination system for site-specific excision of chromosomally located DNA sequences: application for isolation of unmarked *Pseudomonas aeruginosa* mutants. Gene, 1998. **212**(1): p. 77–86.
7. Shi, J., et al., PA5470 Counteracts Antimicrobial Effect of Azithromycin by Releasing Stalled Ribosome in *Pseudomonas aeruginosa*. Antimicrob Agents Chemother, 2018. **62**(2): 10.1128.
8. Shi, J., et al., SuhB is a novel ribosome associated protein that regulates expression of MexXY by modulating ribosome stalling in *Pseudomonas aeruginosa*. Mol Microbiol, 2015. **98**: 370-383.
