# Supplementary material for: Pseudomonas aeruginosa tRNA nucleotidyltransferase Cca controls resistance and tolerance to aminoglycoside antibiotics by regulating the MexXY multidrug efflux pump
Source: Antimicrob Agents Chemother. 2026 Mar 3;70(4):e01653-25. doi: 10.1128/aac.01653-25 (PMC13041342; doi:10.1128/aac.01653-25)
Supplement: Table S3 — Primers used in this study. [file aac.01653-25-s0004.doc]

**Table S3**. Primers used in this study.

| Primer^a^ | Sequence 5’-3’ | Use |
| --- | --- | --- |
| *PA5149.1*-F1 | CGGAATTCTGCCCAGGTAGCTCAGTTGGTAGAG | pUCP20-*PA5149.1* |
| *PA5149.1*-R1 | CGGGATCCTGGTGCCCAGGGACGGAATC |
| *PA4669.1*-F1 | CGGAATTCCAGGGGCGTCGCCAAGC | pUCP20-PA4669.1 |
| *PA4669.1*-R1 | CGGGATCCTGGCAGGGGCGGCTG |
| P*_armZ_*-*armZ*-F | CGGGAGCTCTGCGCCGCTCATTTC | P*_armZ_*-*PA5471.1*-*armZ*-His |
| P*_armZ_*-*armZ*-R | CGGGATCCTCAATGGTGATGGTGATGATGTCGGCAGCACTCCCCAC |
| P*_armZ_*-Δ-*armZ*-UF | CGAGCTCTGCGCCGCTCATTTCCACCG | P*_armZ_*-Δ-*armZ*-His |
| P*_armZ_*-Δ-*armZ*-UR | TCCAATCCATGTGATTGAAACGATGTAACCTCCTTTGCGG |
| P*_armZ_*-Δ-*armZ*-DF | TCAATCACATGGATTGGATTATGGGCAACTACATCAAGCC |
| P*_armZ_*-Δ-*armZ*-DR | CGGGATCCTCAATGGTGATGGTGATGATGTCGGCAGCACTCCCCAC |
| P*_armZ_*-Q3Am-*armZ*-UF | CGAGCTCTGCGCCGCTCATTTCCACCG | P*_armZ_*-Q3Am-*armZ*-His |
| P*_armZ_*-Q3Am-*armZ*-UR | CTGCAAAGGGTAGAGAACTAAATCATCGATGTAACCTCC |
| P*_armZ_*-Q3Am-*armZ*-DF | CATCGATGATTTAGTTCTCTACCCTTTGCAGGC |
| P*_armZ_*-Q3Am-*armZ*-DR | CGGGATCCTCAATGGTGATGGTGATGATGTCGGCAGCACTCCCCAC |
| P*_armZ_*-*PA5471.1*_Q3K_-*armZ*-UF | CGGGAGCTCTGCGCCGCTCATTTC | P*_armZ_*-*PA5471.1*_Q3K_-*armZ*-His |
| P*_armZ_*-*PA5471.1*_Q3K_-*armZ*-UR | CAAAGGGTAGAGAACTTAATCATCGATGTAACCTCCTTTGCG |
| P*_armZ_*-*PA5471.1*_Q3K_-*armZ*-DF | GGTTACATCGATGATTAAGTTCTCTACCCTTTGC |
| P*_armZ_*-*PA5471.1*_Q3K_-*armZ*-DR | CGCGGATCCTCAGTGGTGGTGGTGGTG |
| P*_armZ_*-*PA5471.1*_F4L_-*armZ*-UF | CGGGAGCTCTGCGCCGCTCATTTC | P*_armZ_*-*PA5471.1*_F4L_-*armZ*-His |
| P*_armZ_*-*PA5471.1*_F4L_-*armZ*-UR | CAAAGGGTAGATAACTGAATCATCGATGTAACCTCCTTTGCG |
| P*_armZ_*-*PA5471.1*_F4L_-*armZ*-DF | GGTTACATCGATGATTCAGTTATCTACCCTTTGC |
| P*_armZ_*-*PA5471.1*_F4L_-*armZ*-DR | CGCGGATCCTCAGTGGTGGTGGTGGTG |
| P*_armZ_*-*PA5471.1*_Q3KF4L_-*armZ*-UF | CGGGAGCTCTGCGCCGCTCATTTC | P*_armZ_*-*PA5471.1*_Q3KF4L_-*armZ*-His |
| P*_armZ_*-*PA5471.1* _Q3KF4L_-*armZ*-UR | CAAAGGGTAGATAACTTAATCATCGATGTAACCTCCTTTGCG |
| P*_armZ_*-*PA5471.1*_Q3KF4L_-*armZ*-DF | GGTTACATCGATGATTAAGTTATCTACCCTTTGC |
| P*_armZ_*-*PA5471.1*_Q3KF4L_-*armZ*-DR | CGCGGATCCTCAGTGGTGGTGGTGGTG |
| *PA5149.1*-F2 | CTAGATGCCCAGGTAGCTCAGTTGGTAGAGCAGGGGATTGAAAATCCCCGTGTCGGCGGTTCGATTCCGTCCCTGGGCACCAG | pDN19-*PA5149.1* |
| *PA5149.1*-R2 | AATTCTGGTGCCCAGGGACGGAATCGAACCGCCGACACGGGGATTTTCAATCCCCTGCTCTACCAACTGAGCTACCTGGGCAT |
| *PA4669.1*-F2 | CTAGACAGGGGCGTCGCCAAGCGGTAAGGCAGCAGGTTTTGATCCTGCCATGCGTTGGTTCGAATCCAGCCGCCCCTGCCAG | pDN19-*PA4669.1* |
| *PA4669.1*-R2 | AATTCTGGCAGGGGCGGCTGGATTCGAACCAACGCATGGCAGGATCAAAACCTGCTGCCTTACCGCTTGGCGACGCCCCTGT |
| *cca*-F | AATTCGATCATGCATGAGCTCCCGGCCTGCAAGGTTTCCAG | pUC18T-mini-Tn7T-*cca* |
| *cca*-R | GGGGGATCCACTAGTGAGCTCTCAGGCCTTTCCGCGTTCTTCC |
| *cca*-UF | CGGAATTCGAGGGCGAGGAAGGCTACCC | pEX18Tc-*cca* |
| *cca*-UR | CACCGACCACCACCCAGTCG |
| *cca*-DF | CGACTGGGTGGTGGTCGGTGCTGGAACAGCGGGACTATCCGC |
| *cca*-DR | GCTCTAGACATTCCCTGGCTGCGCATCC |
| P*_Tn7R_* | CACAGCATAACTGGACTGATTTC | Test insertion of pUC18T-mini-Tn7T-*cca* |
| P*_glmS_*_-down_ | GCACATCGGCGACGTGCTCTC |
| q*mexY*-F | CTCTACACCAAGGCCACCAG | qPCR |
| q*mexY*-R | GACGATCTTCAGGCGGTTCT |
| q*mexX*-F | AAGGTGGTCAACCCAAAGGG | qPCR |
| q*mexX-*R | GTTCTCGACGATCACCCACT |
| q*armZ*-F | CTCCGTCGGTCGCTTCTATC | qPCR |
| q*armZ*-R | CTTTTCCAGTTTGTCCGCCG |
| q*rpsL*-F | GTATACACCACCACGCCGAA | qPCR |
| q*rpsL*-R | GTGACCTTCACCACCGATGT |
| q*PA0668.1*-F | AAGGTCTTCGGATTGTAA | qPCR |
| q*PA0668.1*-R | GTGCTTATTCTGTTGGTAA |
| q*PA5471.1*-F | TACCCTTTGCAGGCTGCGC | qPCR |
| q*PA5471.1*-R | GCGGAAGGGCGAAGGAGA |

a: F: forward; R, reverse; U, upstream of specific gene; D, downstream of specific gene; q, qPCR.
